# Temperature-sensitive migration dynamics in neutrophil-differentiated HL-60 cells

**DOI:** 10.1038/s41598-022-10858-w

**Published:** 2022-04-29

**Authors:** Galina Khachaturyan, Andrew W. Holle, Karen Ende, Christoph Frey, Heiko A. Schwederski, Tim Eiseler, Stephan Paschke, Alexandre Micoulet, Joachim P. Spatz, Ralf Kemkemer

**Affiliations:** 1 Department of Cellular Biophysics, Max Planck Institute for Medical Research, 69120 Heidelberg, Germany; 2 Department of Biophysical Chemistry, University of Heidelberg, 69120 Heidelberg, Germany; 3 Mechanobiology Institute, National University of Singapore, 117411 Singapore, Republic of Singapore; 4 Department of Biomedical Engineering, National University of Singapore, 117411 Singapore, Republic of Singapore; 5 School of Applied Chemistry, Reutlingen University, Alteburgstrasse 150, 72762 Reutlingen, Germany; 6 Internal Medicine I, University Clinic Ulm, 89081 Ulm, Germany; 7 General and Visceral Surgery, University Clinic Ulm, 89081 Ulm, Germany

**Keywords:** Cell migration, Cellular motility

## Abstract

Cell migration plays an essential role in wound healing and inflammatory processes inside the human body. Peripheral blood neutrophils, a type of polymorphonuclear leukocyte (PMN), are the first cells to be activated during inflammation and subsequently migrate toward an injured tissue or infection site. This response is dependent on both biochemical signaling and the extracellular environment, one aspect of which includes increased temperature in the tissues surrounding the inflammation site. In our study, we analyzed temperature-dependent neutrophil migration using differentiated HL-60 cells. The migration speed of differentiated HL-60 cells was found to correlate positively with temperature from 30 to 42 °C, with higher temperatures inducing a concomitant increase in cell detachment. The migration persistence time of differentiated HL-60 cells was higher at lower temperatures (30–33 °C), while the migration persistence length stayed constant throughout the temperature range. Coupled with the increased speed observed at high temperatures, this suggests that neutrophils are primed to migrate more effectively at the elevated temperatures characteristic of inflammation. Temperature gradients exist on both cell and tissue scales. Taking this into consideration, we also investigated the ability of differentiated HL-60 cells to sense and react to the presence of temperature gradients, a process known as thermotaxis. Using a two-dimensional temperature gradient chamber with a range of 27–43 °C, we observed a migration bias parallel to the gradient, resulting in both positive and negative thermotaxis. To better mimic the extracellular matrix (ECM) environment in vivo, a three-dimensional collagen temperature gradient chamber was constructed, allowing observation of biased neutrophil-like differentiated HL-60 migration toward the heat source.

## Introduction

Cellular motility is a fundamental process essential for embryonic development, tissue genesis, wound healing, and immune response. The mechanisms of basic cell migration have been studied extensively with many different cell types. In a wide range of epithelial and mesenchymal cells, migration is known to be a multistep cycle. F-actin polymerization at the leading edge creates an organelle-free membrane protrusion, which subsequently, through integrin-mediated attachment, becomes anchored to the substrate. Traction force generated by the actin-myosin complex and cell detachment at the rear end results in forward cell movement. Cell migration can be quantitatively characterized by a number of parameters, including speed, velocity, and directional persistence^1^.

Moving cells have varying degrees of intrinsic directionality. Random migration occurs when the intrinsic positive feedback loop control, stabilizing the leading edge of the cell is weak^2^. Cell speed and directionality can also be influenced by external factors such as ECM structure^3^, composition^4^, or stiffness^5^, application of physical strain^5^ or electrical fields^6^, presence of chemoattractants^7^, and temperature^8^.

Despite the core temperature being 37 °C, temperature is not a constant in the human body. During any kind of illness, the body’s normal response is increase in body temperature. Additionally, temperature gradients exist on both the single cell level^9,10^ and the tissue-scale level^11^. These gradients play an important role in many physiological processes such as metabolism, cell division and gene expression^12^. Chrétien et al. recently demonstrated that mitochondria, the organelle responsible for synthesis of ATP in the cells, are physiologically maintained at 50 °C, approximately 13 °C degrees higher than the average cell temperature^13^. Temperature can also affect vital white blood cell functions, including phagocytic activity in monocytes^14^ and superoxide production in neutrophils^15^. Fever-range high temperatures have been shown to regulate and enhance antigen-specific CD8^+^ T cell differentiation^16^. Furthermore, temperature gradients have been shown to influence embryonic development in Drosophila melanogaster^17^. Mammalian sperm cells also use temperature cues to navigate the female genital tract^18^. Thermal gradients arise in diseased and inflamed tissues as well. Similar to cell chemotaxis, directed cell migration guided by temperature gradients is termed thermotaxis^18^.

As part of the body’s immune system, leukocytes are required to navigate various tissues with alternating temperatures in order to successfully migrate towards an injured or infected site. Neutrophils are the most abundant white blood cells in the human body and are the first cells to respond to infection or tissue damage^19^. Their ability to swiftly detect signals released by damaged host cells or microorganisms and react by polarizing and migrating towards the source is crucial to the body’s initial inflammatory response^20^. The first step in neutrophil activation is rapid F-actin polymerization at the leading edge, polarizing the cell^21^. Many signaling molecules are involved in this step, and a great deal of work has been performed to fully understand the mechanism of neutrophil activation and directional migration^22–24^. External temperature can also affect F-actin polymerization^25^, the diffusion rate of signaling molecules and integrins in neutrophils^26^, cell membrane fluidity^27,28^ and many other processes involved in directed neutrophil migration. To our knowledge, a thorough investigation of temperature-dependent neutrophil migration has not yet been performed.

One of the most common model cell lines used for in vitro studies of neutrophil migration are differentiated HL-60 cells^29–31^. Unlike neutrophils isolated from human blood, which are terminally differentiated, short-lived, and cannot be genetically manipulated, HL-60 cells are immortal and can be transfected^32^. Hauert et al. systematically compared the response of human peripheral blood neutrophils and neutrophil-differentiated HL-60 cells to different chemical stimuli. They showed that changes in cell morphology and chemotaxis occurring in response to chemoattractants and different inhibitors are both quantitatively and qualitatively comparable to those induced in primary neutrophils. Moreover, they found that levels of signaling enzymes and proteins in HL-60 cells upon differentiation are similar to those found in primary neutrophils^33^.

Recently, a number of studies have attempted to characterize neutrophil migration in three dimensions. In vivo, activated neutrophils must migrate through endothelial walls and a number of diverse tissues. Wilson et al. used neutrophil-differentiated HL-60 cells as a model system for investigating actin dynamics during interstitial 3D neutrophil migration using fluorescent labelling, a technique not compatible with primary neutrophils^31^.

In this study, we investigated the response of neutrophil-differentiated HL-60 cells to temperature changes in their environment by analyzing 2D migration in physiologically relevant temperatures ranging between 30 °C and 42 °C. In general, increasing temperature resulted in increases in cell migration speed. Interestingly, despite significant increases in speed, directional persistence length stayed constant throughout normal and hyperthermic temperature ranges. The effect of substrate ECM coating was also analyzed, with high levels of serum protein resulting in faster migration. Fibronectin coating, on the other hand, significantly reduced cell speed. We also investigated the ability of neutrophil-differentiated HL-60 cells to thermotax in both two and three dimensions in response to an external temperature gradient.

## Results and discussion

### HL-60 cell differentiation

HL-60 cells are immortal human blood promyelocytic leukemia cells capable of terminal differentiation into more mature white blood cell types such as neutrophils or monocytes. In our studies, HL-60 cells were differentiated into neutrophil-like cells using 1.3% DMSO supplemented media. Differentiation was induced for 5–7 days, after which cells were resuspended in DMSO-free media. To qualitatively confirm the success of the differentiation into neutrophil-like cells, Giemsa-Wright staining was performed prior to and after the differentiation protocol. Prior to induction, HL-60 cells had rounded nuclei typical of that cell line, whereas the majority of differentiated cells exhibited multi-segmented nuclei characteristic of neutrophils (Supplemental Fig. 1a). Flow cytometry was also used to assess differentiation efficiency, with 86.72% of cells found to be fully differentiated after 5 days of 1.3% DMSO treatment, as measured by CD11b expression (Supplemental Fig. 1c–d).

Non-differentiated HL-60 cells are non-adherent and cultured in suspension. Further confirmation of successful differentiation is the presence of adherent cells. By the third day of differentiation, cells were observed adhering to the bottom of the culture flasks. By the seventh day post-induction, the number of adherent cells increased five-fold (Supplemental Fig. 1b).

### Temperature-dependent attachment and migration speed in differentiated HL-60 cells

Differentiated HL-60 cells were used to investigate neutrophil migration under the effect of physiologically relevant temperatures (30–42 °C). Here, this thermal range is divided into 3 categories: hypothermic (30–35 °C), normal (35–39 °C), and hyperthermic (39–42 °C).

**Figure 1:**
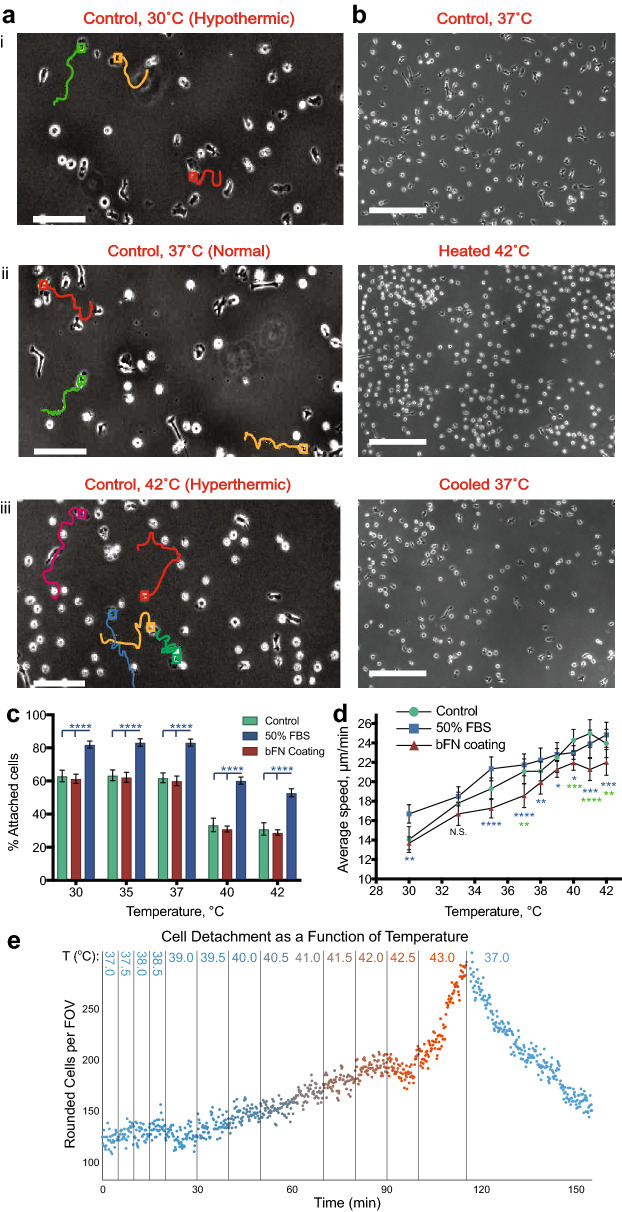
Temperature-dependent attachment and migration dynamics of differentiated HL-60 cells.** (a)** Phase contrast images of control cell tracks at 30 °C (hypothermic), 37 °C (normal) and 42 °C (hyperthermic). A large number of cells detached under hyperthermic conditions. Scale bars: 100 µm. **(b)** Phase contrast images of cells at control conditions at 37 °C, heated at 42 °C and cooled back to 37 °C. Detached cells did not appear to be dead or apoptotic, as reduction of the temperature back to 37 °C resulted in cell reattachment to the substrate. Scale bars: 200 µm. **(c)** Cell attachment at different seeding conditions. In control samples under hyperthermic conditions, ~ 50% of the initially attached cells detached. 100 µg/ml bovine fibronectin (bFN) coating did not improve cell attachment compared to the control condition (no coating, 10% FBS), whereas a fivefold increase in FBS (50% FBS) in the media resulted in a significantly higher (~ 20%) number of attached cells throughout the entire (30–42 °C) temperature range. **(d)** The average cell speed ± 95% CIs of differentiated HL-60 cells at each experimental temperature. Cell speed increased linearly with increasing temperature for all conditions. Cells seeded in the presence of 50% FBS did not show any significant increase in average speed. 100 µg/ml bFN surface coating significantly reduced average cell speed at normal and hyperthermic temperatures. 39 and 78 cells were tracked for all conditions in hypothermic and normal/hyperthemic temperature ranges, respectively. At 39 and 40 °C on bFN coating, 117 cells were tracked. **(e)** The number of detached cells, as measured by automatically identified rounded cells, as a function of temperature. Data was captured every 10 s for 2.5 h. For **(a–d)** 2 independent experiments were performed. *P < 0.05, **P < 0.01, ***P < 0.001, ****P < 0.0001, N.S. not significant.

Differentiated HL-60 cells cultured on untreated glass bottom petri dishes remained motile throughout the entire temperature range applied (Fig. 1a). Elevated temperatures in the hyperthermic range significantly reduced the amount of adherent cells. At 40 °C, ~ 50% of previously attached cells were observed floating in the media (Fig. 1aiii). Detached cells are not dead or apoptotic, as reduction of the temperature back to 37 °C resulted in reattachment to the substrate (Fig. 1b,e, Supplemental Video 1). Additionally, relative cell viability for differentiated HL-60 cells was determined by measuring ATP of viable cells using a CellTiter-Glo assay, indicating no significant changes as a function of temperature over 45 min (Supplemental Fig. 2). To improve cell adhesion at higher temperatures, elevated serum concentration (50% FBS) and a 100 µg/mL fibronectin (bFN) surface coating were both tested. Fibronectin coating did not improve cell attachment in any of the temperature regimes (Fig. 1c). However, higher serum concentration resulted in a 20–30% increase in cell attachment for the entire range of temperatures analyzed. This could be due to high levels of surface-adsorbable ECM proteins commonly found in serum^34^, which provide an increased number of binding ligands at the cell-surface interface. As the elevated levels of serum were maintained throughout the experiment, it is also possible that the resulting increase in soluble protein concentration in the media also encouraged attachment and cell migration.

The mean speed of differentiated HL-60 cells varied between different conditions and as a function of temperature. For all three seeding conditions, the mean migration speed increased nearly linearly with increasing temperature, with each increasing degree Celsius causing a commensurate increase in cell speed of approximately 0.87 µm/minute for control surfaces, 0.65 µm/minute for 50% FBS, and 0.71 µm/minute for fibronectin-coated surfaces. No significant difference in mean speed was observed between cells cultured in media containing 10% FBS or 50% FBS. Fibronectin coating significantly reduced cell speed (Fig. 1d). This decrease is likely due to the stronger adhesion promoted by fibronectin, resulting in an altered cell migration speed^4^. Increases in cell speed could be related to actin polymerization acceleration corresponding to increases in temperature, as described by Rosin et al.^25^. It has also been shown that F-actin concentration in PMNs increases in response to external signals such as chemoattractants. Howard et al.^35^ showed that both chemoattractant signaling and the application of increased temperature are sufficient to cause an increase in F-actin concentration in human blood neutrophils. They showed that between temperatures of 30 °C and 40 °C, F-actin concentration increases linearly, resulting in doubling by 40 °C. This supports our hypothesis that linear increases in the average speed of neutrophil-like differentiated HL-60 cells could be a result of higher F-actin polymerization rates and subsequent increases in absolute F-actin levels as a result of increasing temperature.

### Temperature-dependent effects on differentiated HL-60 cell directionality

**Figure 2:**
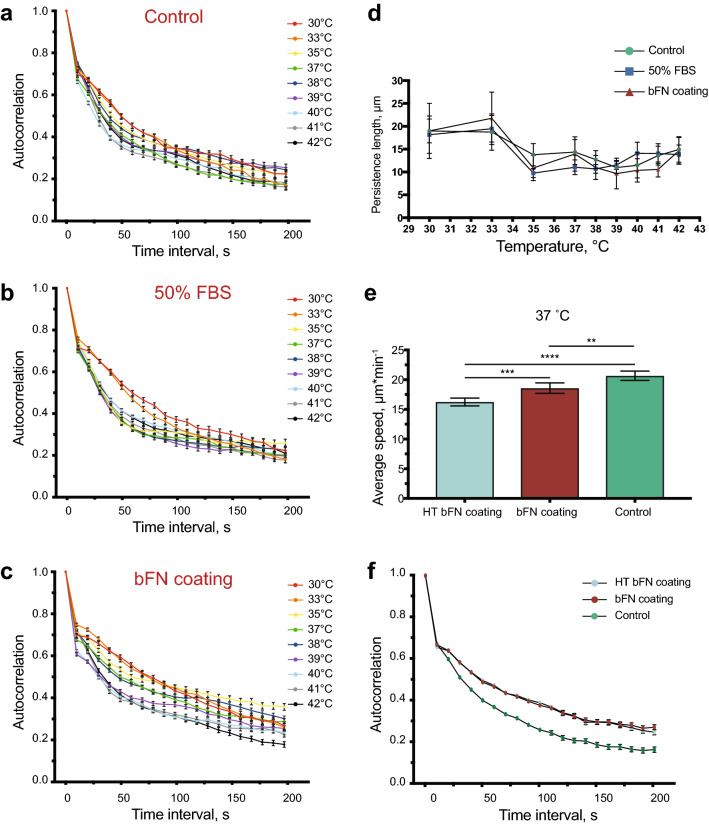
Temperature- and ECM-dependent direction correlation of differentiated HL-60 cell migration.** (a–c)** Direction autocorrelation analysis of differentiated HL-60 cells under 3 different conditions (control, 50% FBS and bFN coating) and 9 different temperatures between 30 and 42 °C. At hypothermic temperatures, directional autocorrelation peaks for all conditions. On bFN coating, cells maintain directionality for longer time periods. Error bars represent S.E.M. **(d)** Persistence length (µm) of differentiated HL-60 cells was calculated by multiplying the persistence time by average cell speed. The persistence time was evaluated by fitting Eq. (1) to the autocorrelation curves. At normal and hypothermic conditions, the persistence length was preserved. Error bars represent 95% CIs. **(e)** Average cell speed of differentiated HL-60 cells without coating (control), on 100 µg/ml bovine fibronectin coating (bFN coating), and on heat-treated bovine fibronectin coating (HT bFN coating). Heat treatment of the coating significantly reduced average cell speed. **(f)** Direction autocorrelation of differentiated HL-60 cells on control, bFN and HT bFN coatings at 37 °C. Heat treatment of bovine fibronectin did not influence cell persistence. Error bars are S.E.M. For all: 2 independent experiments were performed. **P < 0.01, ***P < 0.001, ****P < 0.0001.

Direction autocorrelation was calculated in differentiated HL-60 cells for 3 conditions and 9 temperatures using an algorithm published by Gorelik et al.^1^ (Fig. 2a–c). This describes the correlation in migration path orientation over increasing time intervals. Direction autocorrelation coefficients are computed by averaging the cosine of all angle differences over increasing time intervals between two migration vectors that have been normalized to the same length. These coefficients are then averaged for all cells tracked over all pairs of time points and plotted against applied time intervals. The resulting curves can be understood as follows: The steeper the decay of the plotted curves, the more frequently the cells turn in different directions.

To quantitatively approximate the persistence length of the cells, autocorrelation curves were fitted to Eq. (1) to yield the persistence time $\tau$, which is the time period below which directional orientation remains correlated. Average persistence length was then estimated by multiplying persistence time by the average speed at a given temperature. The three substrate coating conditions did not affect cell average persistence length significantly at any given temperature (Fig. 2d). Interestingly, while cells exhibit longer persistence lengths at hypothermic temperature regimes, persistence length remained similar throughout normal and hyperthermic regions. For all three surface coatings, cells cultured between 35 °C and 42 °C had consistent persistence lengths between 9.8 and 14.9 µm.

Houk et al. have shown that membrane tension plays a pivotal role during directed neutrophil migration by restricting the signaling molecules to the leading edge of the cells^23^. Accordingly, decreases in membrane tension cause a loss of polarity. Increases in temperature results in higher membrane fluidity, which in turn reduces membrane tension and can cause cells to stop and turn more frequently, resulting in a decrease in cell persistence time. On the other hand, as previously mentioned, increases in temperature also increase the rate of F-actin polymerization and the total amount of F-actin in the cell. Based on our results, we presume that these two factors combine to balance each other out, causing cells to have a constant persistence length at temperatures higher than 33 °C. Furthermore, even in conditions that cause a significant reduction in cell speed, like fibronectin coating, persistence length remains constant. There are many examples of cells exhibiting correlated migration even in the absence of external directional signals. Li et al. ^36^ showed that *Dictyostelium discoideum*, an amoeba exhibiting fast motility and chemotactic behavior similar to neutrophils, are capable of directed motion by “remembering” their last turn, even in the absence of an external signal. This improves their search efficiency relative to random walk and allows them to search more territory. Similarly, we propose that adherent immune cells have an innate persistence, unaffected by ECM composition or temperature, allowing them to navigate microenvironments with diverse protein compositions and thermal conditions. We theorize that the preservation of persistence length confers an advantage to immune cells migrating at higher temperatures, as they are able to cover more ground in search of their target independent of extracellular matrix composition. The effect of elevated temperatures on membrane fluidity may also be favorable to immune cells, as it allows for faster adaptation of migration direction in response to obstacles.

The relationship between cell persistence time and cell speed has been examined in a number of cell models^37^. In general, as cell speed increases, cell persistence increases concomitantly, up to a plateau value^37^. Here, when increases in speed are a function of increases in temperature, we have observed the opposite effect, as persistence time decreases with increasing temperature (Supplemental Fig. 3). This is likely due to the aforementioned temperature-dependent changes in membrane fluidity.

To determine whether increases in temperature were altering the fibronectin layer, an experiment utilizing pre-heated fibronectin was performed. Fibronectin-coated glass was heated for 1 h at 41 °C. After cooling to 37 °C, differentiated HL-60 cells were seeded onto the heat-treated fibronectin and time-lapse movies were taken at 37 °C for 1 h. The heat treatment of the coating significantly reduced average cell speed compared to non-treated fibronectin (Fig. 2e). As fibronectin is known to unfold and expose additional binding ligands^38^, it is possible that the heat treatment encourages irreversible unfolding or altered refolding. Interestingly, autocorrelation was not affected by fibronectin heat treatment, further supporting the hypothesis that intrinsic cell persistence is not affected by the ECM composition (Fig. 2f).

### PMA-induced oxidative burst in differentiated HL-60 cells

To determine if differentiated cells were capable of respiratory burst at elevated temperatures, the oxidation of dihydrorhoadmine (DHR123) was tested via flow cytometry in the presence of phorbol 12-myristate 13-acetate (PMA) (Supplemental Fig. 4a). For all temperature conditions, the percentage of DHR123-positive cells was significantly increased in response to PMA treatment. This effect was most noticeable at 30 and 37 °C, where a four-fold increase was observed. At 42 °C, only a two-fold increase in DHR123 positive cells occurred (24% vs 48%), proving that differentiated HL-60 cells remain effective at elevated temperatures, although not to the same level found at hypothermic or homeostatic temperature conditions (Supplemental Fig. 4b).

### Thermotaxis in differentiated HL-60 cells

**Figure 3:**
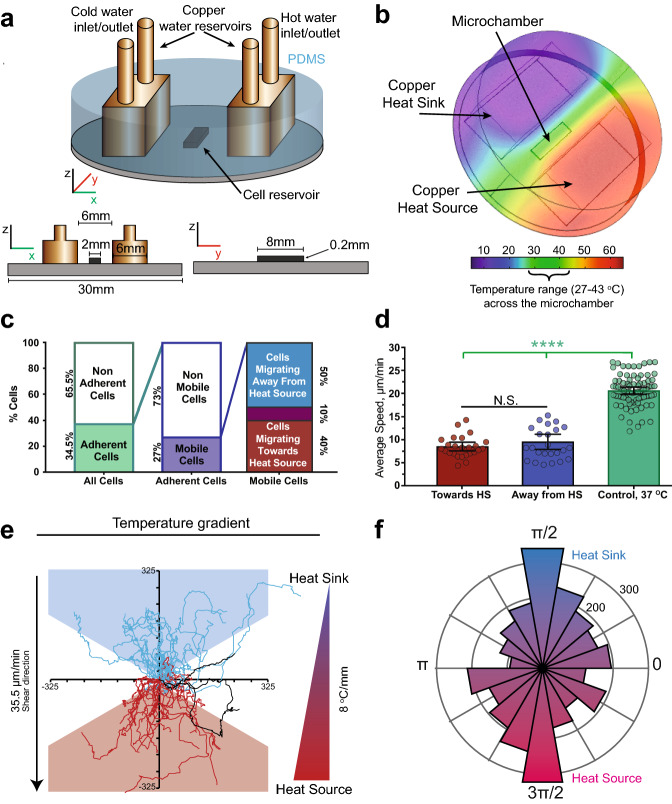
Differentiated HL-60 cell thermotaxis in response to temperature gradients.** (a)** Schematic representation of the microfluidic temperature gradient chamber with cross sections in X and Y directions. Copper reservoirs were used as a heat source and a heat sink, with their temperatures held constant at 65 °C and 5 °C, respectively. (**b**) 3D Volume temperature gradient distribution computed by COMSOL Multiphysics. A 27–43 °C temperature gradient is generated along the 2 mm wide microchannel. (**c**) Temperature gradient effects on cell adhesion and migration. After application of the gradient, ~ 40% of cells remained attached. 72% of the attached cells were non-mobile. Of the mobile cells, approximately 50% migrated towards the heat sink (negative thermotaxis) and 40% migrated towards the heat source (positive thermotaxis). (**d**) Differentiated HL-60 cell speed in the presence of the temperature gradient. 25 cells in each direction were tracked. No significant differences in cell speed were found between those moving towards the heat source and those toward the heat sink, but both were approximately 50% slower than those in a constant 37 °C environment. (**e**) Migration tracks of 50 cells migrating both towards and away from the heat source. Cells that migrated at an angle of 60˚ or less with respect to temperature gradient direction (in this case the Y axis) are considered to have directed migration towards either the heat source or sink (red and blue triangles). 10% of cells showed non-directed migration. (**f**) Polar histogram of the relative angles from cell step to cell step calculated for 50 cells tracked under temperature gradient. Small steps less than 2 µm were disregarded. For all: 2 independent experiments were performed. Error bars represent 95% CIs of the data. ****P < 0.0001, N.S. not significant.

The ability of the neutrophil-differentiated HL-60 cells to sense temperature gradients and thermotax in response was investigated on glass surface using a microfluidic temperature gradient system (*µ*TGS) (Fig. 3a). Computations performed using COMSOL Multiphysics showed the temperature gradient along the whole microchamber was uniform with a range from 27 to 43 °C (Fig. 3b).

Differentiated HL-60 cells were injected into the microchamber at a concentration of ~ 150 cells/mm^2^. The temperature gradient in the microchamber resulted in heat transfer by convection, which caused media flow from the heat sink to the heat source above the glass surface. Up to 60% of cells detached in the microchamber, possibly due to the combination of shear flow and elevated temperatures in regions of the chamber (Fig. 3c). Of the cells that remained adherent, the majority (~ 70%) either remained in place or migrated very little from their position. These cells were considered to be non-mobile cells (Fig. 3c). The remaining mobile cells were classified into three subpopulations based on their final positions within equally-sized 120˚ polar divisions: cells migrating toward the heat source (positive thermotaxis), cells migrating toward the heat sink (negative thermotaxis), and cells moving perpendicular to the temperature gradient (Fig. 3c,e). Based on these divisions, 40% of cells were found to exhibit positive thermotaxis and 50% of cells negative thermotaxis. Interestingly, approximately 10% of motile cells finished in the perpendicular subpopulation, compared to an expected value of 33% for unbiased migration (Fig. 3c). To quantify this tendency, the relative angles between each cell step greater than 2 µm (*θ*) were calculated and plotted as a polar histogram, revealing significant migrational bias along the temperature gradient (Fig. 3f).

The migration speed of motile differentiated HL-60 cells was also evaluated. Independent of migration direction, the average cell speed in the presence of the temperature gradient was approximately half the average speed of cells in a constant 37 °C environment. No significant difference was found between the average speed of positively or negatively thermotaxing cells (Fig. 3d).

### Differentiated HL-60 cell migration under shear flow

To determine whether directed migration towards the heat source was affected by the gradient-generated fluid shear stress, additional migration studies of differentiated HL-60 cells under laminar flow were performed. To approximate the flow rate created in the temperature gradient chamber, the average floating speed of 60 detached cells at different positions was calculated (35.5 µm/min). Using a commercial microchamber flow slide with the same height as the temperature gradient microchannel and a syringe pump, it was established that flow rates of 30 µL/hour results in an average detached cell floating speed of 37 µm/min, i.e. similar to that observed in the temperature gradient chamber. A higher 40 µL/h flow rate was also applied, resulting in a higher shear flow rate that doubled the floating speed of detached cells (80.5 µm/min).

**Figure 4:**
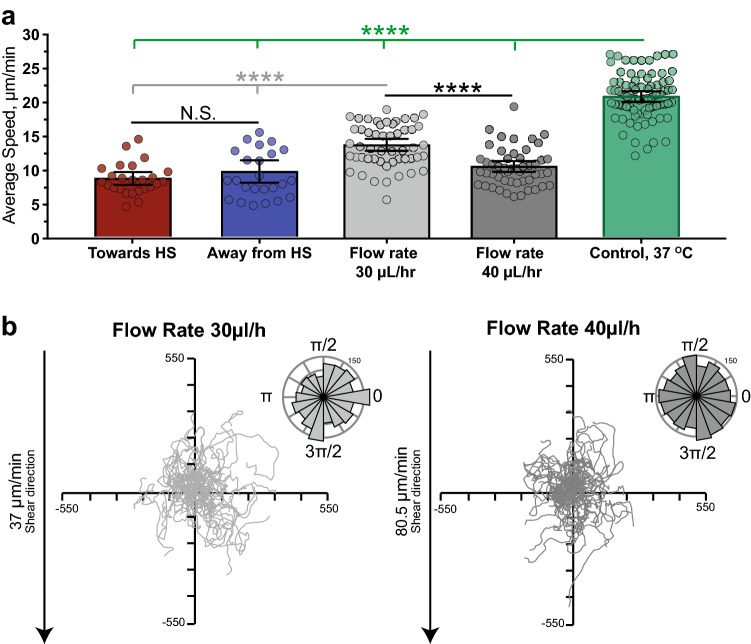
Fluid shear flow of a similar magnitude to that produced by temperature gradients does not induce directed migration.** (a)** Cell speed of differentiated HL-60 cells in the presence of a temperature gradient and under laminar flow of 30 and 40 µL/h. The average speed of 50 cells in total was evaluated for each condition. In the 2 temperature gradient chamber, 25 cells in each direction were tracked at a sampling interval of 15 s. No significant difference between migration speed of cells moving towards either heat source or sink was found by Welch’s t-test. When compared with cell speed in a constant 37 °C environment (sampling interval 10 s), cell speed was reduced by approximately 50%. Application of shear flow also resulted in significant cell speed reduction. Lower cell velocities could partially be an artifact of the lower cell tracking rate of 60 s, but the significant differences in cell speed under lower and higher flow rates indicate that cell migration speed has an inverse relationship to flow rate. (**b**) Migration tracks and polar histograms of θ for 50 cells migrating under laminar flow conditions. Application of 30 and 40 µL/hour flow rates did not result in directional cell migration.

The speed with which the differentiated cells migrated under the applied flow conditions was significantly lower than the speed of the control cells at 37 °C without any external stimuli (Fig. 4a). Although 40 µL/hour flow resulted in slower cell migration than 30 µL/hour flow, neither flow rate was sufficient to induce directional bias (Fig. 4b). This suggests that cells in the temperature gradient microchannel are unlikely to be affected by the gradient-induced convective flow, and thus, their directional bias is a result of the temperature gradient.

While primary neutrophils have been shown to exhibit positive thermotaxis^39^, negative thermotaxis has not been observed. Based on our previous observations, a significant number of the differentiated HL-60 cells detach in response to increasing temperatures (Fig. 1b). Hence, the “negative” thermotaxis of the second cell population could be attributed to cell migration from a weakly-adherent surface to strongly-adherent surface in a manner similar to haptotaxis. This is supported by our previous experiments showing a decrease in cell migration speed on heat-treated fibronectin (Fig. 2e).

### Thermotaxis in differentiated HL-60 cells in 3D collagen gels

**Figure 5:**
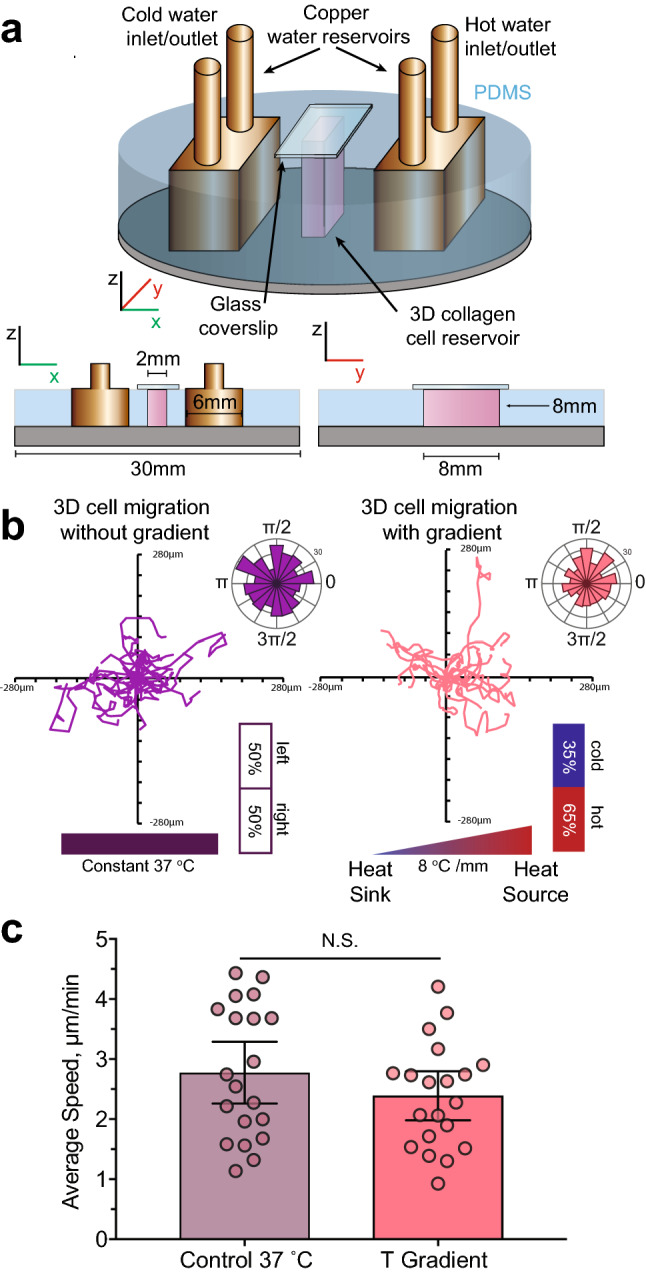
Differentiated HL-60 cell thermotaxis in response to temperature gradients in 3D microchambers.** (a)** Schematic representation of the 3D microfluidic temperature gradient chamber with cross sections in X and Y directions. Copper reservoirs were used as a heat source and a heat sink, with their temperatures held constant at 65 °C and 5 °C, respectively. The microchamber was filled with a collagen scaffold containing cells. (**b**) Migration tracks and polar histograms of θ for 20 cells each, migrating in 3D collagen matrix with and without temperature gradients. Application of the temperature gradients in 3D revealed a higher percentage of cells migrating towards the heat source. (**c**) Cell speed of differentiated HL-60 cells in a 3D collagen matrix at a control temperature of 37 °C and in the presence of the temperature gradient. The average speed of 20 cells in each condition was evaluated. For all: Error bars represent 95% CIs of the data. 2 independent experiments were performed. N.S. not significant.

Cell migration on 2D surfaces differs considerably from migration in the 3D microenvironment in vivo. To better mimic in vivo confinement, a new 3D temperature gradient chip was produced featuring a 3D cell reservoir (Fig. 5a). 20 cells were tracked at both a constant 37 °C and under temperature gradient conditions equal to those produced in the 2D microchamber (8.0 °C/mm). In the absence of the gradient, cells exhibited migration without directional bias (Fig. 5b). When the temperature gradient was applied, 65% of the tracked cells migrated towards the heat source. Cells in the 3D microchamber migrated much slower than cells on 2D surfaces, in agreement with the literature^40^, with no significant difference found between cells at a constant temperature and those in the temperature gradient (Fig. 5c).

## Conclusions

One of the four signs of inflammation is increased temperature in the affected tissue^41^. Neutrophil recruitment and subsequent migration is a complex process guided by the combined effort of many different extracellular cues, including chemical gradients, shear flow and possibly changes in temperature. Díaz et al.^42^ showed that fever-range hyperthermia combined with low pH in the environment, which is often present in inflamed and cancerous tissues, significantly increases neutrophil survival. In our study, we systematically analyzed the role of temperature and ECM on neutrophil adhesion, migration speed, and directionality using differentiated HL-60 cells as a model cell line. We found that differentiated HL-60 cells have a constant persistence length at normal and hyperthermic temperatures, regardless of their extracellular ligand environment. This concept is illustrated in Supplemental Fig. 5, which shows how cells with constant persistence length can be more effective at directed migration at higher temperatures. At low temperatures, cells make fewer turns, maintaining their speed and persistence for longer periods of time. At higher temperatures, due to changes in membrane fluidity, cells turn more frequently and have shorter persistence times. However, due to changes in cell velocity, persistence length is preserved, which could be advantageous to immune cells migrating at higher temperatures, as they are able to cover more ground in search of their target independent of extracellular matrix composition. The ability of neutrophils to detach and survive in response to temperature increases found in inflammation sites may also prime them to more effectively migrate through dense ECM, as previous work has shown that confinement induces a fast, adhesion-independent migration mode in dendritic cells^43^.

While any connections between these findings and clinical observations will need to be assessed more critically in the future, the connection between temperature and neutrophil migration and effectiveness is intriguing. Respiratory viral infections have been shown to be more prevalent in the winter^44^, and COPD patients specifically have been found to experience a 0.8% increase in COPD exacerbation with every 1 °C decrease in air temperature^45^. While the epidemiology of temperature-dependent infection is complex^46^, the concept of temperature playing a role in the ability of immune cells to migrate towards sites of infection or inflammation can have important implications on public health in the future.

We also looked at the ability of HL-60 neutrophils to sense and react to temperature gradients both on 2D glass surfaces and in 3D collagen gels. Our experiments revealed that in 2D, motile cells can be divided into two subpopulations exhibiting “positive” (migration towards heat source) and “negative” thermotaxis (migration towards heat sink). Primary neutrophil thermotaxis has been reported^39^, with two subpopulations of thermotactic cells identified. The first was capable of reversing course after the thermotactic stimulus was reversed, while the other continued migration in the same direction. However, this phenomena has not been investigated further since its original observation in 1982. Hence, our findings on 2D surfaces show that neutrophil differentiated HL-60 cells can be a valuable tool for investigating immune cell thermotaxis, a complex process that is not yet well understood.

## Materials and methods

### HL-60 cell culture and differentiation

HL-60 cells were obtained from DSMZ (Braunschweig, Germany) and kept in cell culture at a maximum concentration of 10^6^ cells per milliliter. For differentiation purposes, cells (passage numbers 6–9) were cultured with an initial concentration of 10^5^ cells per milliliter in the presence of 1.3% DMSO (Sigma Aldrich, Steinheim, Germany) in cell culture media (RPMI 1640 + GlutaMax) supplemented with 10% heat inactivated fetal bovine serum (FBS) and 1% Penicillin–Streptomycin antibiotics. Media and all supplements were obtained from Gibco (Frankfurt, Germany). On day 2, cells were centrifuged, counted, and cultured again in presence of 1.3% DMSO with a cell concentration of 10^5^ cells per milliliter. On day 4 the media was changed. On days 5–7 cells were sufficiently differentiated and could be used for experiments.

### Giemsa-Wright staining

Giemsa-Wright staining was performed according to the manufacturer’s instructions (Sigma Aldrich). Briefly, 10^5^ cells in 300 μL media were spun onto a Cytospin slide and subsequently fixed with methanol for 15 min. After drying, slides were incubated with 500 µL of Wright solution for 2 min, rinsed with dH_2_O, dried again, and incubated with a 1:20 Giemsa solution diluted with dH_2_O for 1 h. Afterwards, the slides were thoroughly washed with dH_2_O and dried. Imaging was performed using a Zeiss Axiovert200M microscope (Oberkochen, Germany), a Plan-Apochromat 63 × oil objective (Zeiss) and an AxioCam IC camera (Zeiss).

### Cell imaging and image analysis

Differentiated HL-60 cells (days 5–7) were centrifuged at 1000 g for 5 min, the supernatant was removed, and the cells were resuspended in corresponding RPMI-1640 media not containing DMSO. 200,000–1,000,000 cells in 250 µL media were placed in sterile 20 mm or 35 mm glass-bottomed petri dishes (Greiner Bio-One, Frickenhausen, Germany) and incubated for 1 h at 37 °C and 5% CO_2_. Afterwards, in order to remove as many non-adherent cells as possible, media was aspirated and the petri dishes were washed 3 times. After addition of 2 mL of fresh 37 °C-heated media, the petri dishes were placed on a heating plate and time-lapse movies were collected.

Three different seeding conditions were used for experiments with static temperatures: control, bovine fibronectin (bFN) glass coating, and 50% FBS. Cells seeded on the glass surface of the petri dish without any coating and with media containing 10% FBS was used as a control. For bFN experiments, the petri dish was incubated with 100 µg/mL bFN (Gibco) for 2 h at room temperature and subsequently washed with PBS 3 times for 5 min each. Afterwards, they were filled with media containing 10% FBS and stored until cell seeding. For the third seeding condition, a fivefold increase of FBS in the media compared to the control was used for the duration of the experiment.

After cell seeding, cells were placed on a transparent thermoplate (Tokai Hit, Model TP-KI05-60, Thermofisher, Darmstadt, Germany), which was then placed on the stage of an Axiovert200M microscope, equipped with an incubation chamber to maintain an environment of 5% CO_2_, 37 °C, and high humidity. Time-lapse movies were taken with a 10 × objective (EC Plan-Neofluar, Zeiss) every 10 s. Cells were heated from 30 to 42 °C. During this process, the temperature was held at 30 °C, 33 °C, and 35 °C for 1 h each and at 37 °C for 30 min. Starting with 37 °C, the temperature was changed every 10 min by 0.5 °C until a final temperature of 42 °C. The temperature change of the plate caused thermal expansion, which resulted in gradual defocusing of the images. To address this, imaging was shortly paused at 39 °C for focus adjustment.

To assess cell attachment at different seeding conditions and temperatures, the number of attached and floating cells in 10 positions (each with area of 0.6 mm^2^) from 2 independent experiments was counted. To assess the effects of temperature on cell velocity, for each condition and each temperature, 39–78 cells were manually tracked for 8 min (50 frames) using Fiji’s Manual CellTracker plug-in^47^. At 39 °C and 40 °C on bFN coating, 117 cells were tracked. For the lower temperatures (30–37 °C), videos taken from the last 10 min of each corresponding temperature were analyzed. The instantaneous speed of each cell was then calculated and averaged. The mean speed results presented in this work are averaged from the number of cells tracked for each specific condition at a given temperature.

### CD11b flow cytometry of HL-60 cells

HL-60 differentiation efficacy was evaluated by flow cytometry against CD11b. 100,000 differentiated HL-60 cells were resuspended in 100 µl FACS buffer (PBS + 5% FCS) and incubated with CD11b-FITC (BD Bioscience, # 562793) or FITC-Mouse IgG1, κ Isotype (BD Bioscience, # 562793) antibodies together with DAPI for 30 min on ice. After washing cells twice with FACS buffer, cells were analyzed for FITC-fluorescence using a LSRII-Special Order System (BD Bioscience) by gating on DAPI negative cells, living cells. Conditions: Differentiated HL-60 (CD11b), differentiated HL-60 (isotype), differentiated HL-60 (unstained). Undifferentiated cells served as negative control. 10,000 cells per condition were analyzed. Overlay histograms and quantitative analysis were performed using Flowing Software 2.51, Turku Center for Biotechnology, Finland.

### Neutrophil Respiratory Burst Assays at different temperatures

The ability of differentiated HL-60 cells to perform an oxidative burst at 30, 37 and 42 °C was evaluated by flow cytometry using the Neutrophil Respiratory Burst Assays Kit (Cayman Chemicals, # 601130) with 100,000 cells according to the manufacturer’s description. In brief, 100,000 differentiated HL-60 cells were resuspended in 100 µl assay buffer and labeled with DHR123 dye for 15 min at 37 °C in a cell culture incubator. Subsequently, the respiratory burst was induced by stimulation with 200 nM PMA either at 30, 37 or 42 °C for 45 min. Cells were washed twice in assay buffer and then analyzed using a LSRII-Special Order System (BD Bioscience) in the PE channel. Differentiated unstained and non-differentiated cells which were also subjected to burst induction at 37 °C served as respective controls. Three independent differentiations and 10,000 cells per condition were analyzed. Overlay histograms and quantitative analysis were performed using Flowing Software 2.51 (Turku Center for Biotechnology, Finland).

### CellTiter-Glo® 2.0 Cell Viability Assay

In order to control for relative cell viability after incubating cells in the neutrophil respiratory burst assays at 30, 37 and 42 °C for 45 min, we have performed a CellTiter-Glo® 2.0 Cell Viability Assay according to the manufacturer’s description. Three independent differentiations were analyzed with three technical replicas per condition. 20,000 differentiated and un-differentiated HL-60 cells were seeded in 100 µl assay buffer in white-wall-96-well plates (Greiner Bio One, #655088) and incubated at the respective temperatures as indicated. Subsequently cells were lysed by adding 100 µl of CellTiter-Glo reagent as indicated by the manufacturer. ATP-dependent luminescence from viable cells was determined using a Tecan M200 pro plate reader with 1000 ms integration time. Data is shown as relative light units (RLU).

### 3-D cell culture

Differentiated HL-60 cells were cultured in Collagen I hydrogel scaffolds as described by Shin et al.^48^. Briefly, 20 µL of 10 × DPBS (Gibco) was thoroughly mixed with 11 µL of 0.5 N NaOH solution (Sigma-Aldrich), 133 µL of 3 mg/mL rat tail Collagen I stock solution (Gibco) and 36 µL of cell suspension (200,000 cells overall) for a final concentration of 2.0 mg/mL collagen. All ingredients and vials were precooled and hydrogel solution preparation was performed on ice.

### Autocorrelation analysis

To evaluate differentiated HL-60 cell persistence at different temperatures, autocorrelation analysis was performed on the tracked data of the cells. Autocorrelation was computed over 1/3 of the track length for each condition and temperature using the DiPer macro provided by Gorelik et al.^1^. Exponential fitting of the autocorrelation data was done using the following equation:

1 $$ae^{ - t/\tau } + c$$

where $\tau$ is the persistence time. The average persistence length was then calculated by multiplying the persistence time by the average speed at each temperature.

### Temperature gradient chamber fabrication

Temperature gradient chambers were manufactured based on a modification of the system described in Das et al.^49^, using ridged Plexiglas molds with ridge dimensions of 8.0 × 2.0 × 0.2 mm^3^ (l:w:h) (Fig. 3a). As a heat source and heat sink, rectangular hollow copper reservoirs (18:6:8 mm^3^) were used with inlets and outlets to supply heated and chilled water. These were attached to the mold with screws parallel to the microridge. 1:10 PDMS 184 (Dow Corning, Midland, MI, United States) was poured in the mold and subsequently cured at 110 °C for 40 min. To inject cells into the PDMS microchamber, 2 holes with 1 mm diameter were punched into the PDMS. The PDMS chips were bound to a glass coverslip via oxygen plasma treatment of both the glass and the PDMS. The copper reservoirs were then detached from the mold and inserted into the PDMS microchamber. The temperature gradient was initiated by supplying hot and cold water to the copper reservoirs, continuously exchanged using two peristaltic pumps.

### Temperature gradient analysis

In order to assess the temperature gradient applied to the cells, Comsol Multiphysics 5.1 3D simulations were performed for the geometry used in the experimental setup (Stockholm, Sweden). To simplify the computation, the copper reservoirs were considered to be solid blocks with a constant temperature corresponding to the temperature used during experiments (65 °C for the heat source and 5 °C for the heat sink). Heat transfer in solids mode was applied for the analysis.

### Temperature gradients

Temperature gradient was created by continuously supplying hot and cold water into the copper reservoirs. The temperature of the supplied water was kept constant via a heating plate and an ice bath. A thermocouple was attached to each copper reservoir to monitor the temperature of the reservoirs for the duration of the experiments.

The experiments were performed by injecting cells into the microchamber at a concentration of 150 cells/mm^2^ and imaging every 15 s. Prior to temperature gradient initiation, cells were imaged for 30 min at 37 °C. To achieve the desired temperature gradient range of 27–43 °C, the hot and cold sources were supplied with 65 °C and 5 °C water, respectively. After gradient initiation, cells were imaged for 40 min. 50 cells in total were tracked. The mean cell speed was then calculated as described above.

### Shear flow assay

Channel slides with dimensions of 50 × 5 × 0.2 mm^2^ ((l:w:h), Ibidi GmbH, Mastinsried, Germany) were used to investigate differentiated HL-60 cell behavior under shear flow. The flow rate, controlled by syringe pumps (Pump11Elite, Harvard Apparaturs, USA), was kept at either 30 µL/hour or 40 µL/hour. Media was injected with 1 mL syringes (Omnifix®-F, B.Braun, Germany) connected by a cannula (Sterican®, 0.4 × 20 mm^2^, BL/LB, B.Braun, Germany) and PTFE-tubing (0.4 × 0.9 mm^2^, Bola, Germany). The cells were kept under constant flow for 1 h and imaged at 1 min intervals. For each of the two flow conditions, 2 independent experiments were performed. In total, 50 cells from at least 5 different chamber positions were tracked.

### Statistics

Autocorrelation data is presented as mean ± SEM. All other data is presented as mean ± 95% confidence intervals (CIs). Statistical analysis was performed in GraphPad Prism (San Diego, CA, USA). Significance was evaluated where applicable either by standard unpaired one-way ANOVA, unpaired two-way ANOVA or by unpaired Welch’s t-test. The normality of the t-test was assessed using the D’Agostino & Pearson normality test.

### Supplementary material

See Supplemental Fig. 1 for confirmation of HL-60 cell differentiation, Supplemental Fig. 2 for HL-60 cell survival as a function of temperature, Supplemental Fig. 3 for the relationship between temperature dependent cell migration speed and persistence time, Supplemental Fig. 4 for results of oxidative burst experiments, and Supplemental Fig. 5 for schematics describing the relationship between cell speed, persistence time, and persistence lengths.

## Supplementary Information

Supplementary Video 1.

Supplementary Information 1.

## Data Availability

The datasets generated during the current study are available from the corresponding author on reasonable request.
