# Supplementary material for: Temperature-sensitive migration dynamics in neutrophil-differentiated HL-60 cells
Source: Sci Rep. 2022 Apr 29;12:7053. doi: 10.1038/s41598-022-10858-w (PMC9054779; doi:10.1038/s41598-022-10858-w)
Supplement: Supplementary file 2 — Supplementary Information 1. [file 41598_2022_10858_MOESM2_ESM.pdf]

Supplementary data

Temperature-sensitive migration dynamics in neutrophil-differentiated HL-60 cells

Galina Khachaturyan[1,2], Andrew W. Holle[1,3,4], Karen Ende[5], Christoph Frey[1,2], Heiko A. Schwederski[5], Tim Eiseler[6], Stephan Paschke[7], Alexandre Micoulet [1,2], Joachim P. Spatz[1,2], Ralf Kemkemer[1,5 *]

[1] Department of Cellular Biophysics, Max Planck Institute for Medical Research, 69120 Heidelberg, Germany

[2] Department of Biophysical Chemistry, University of Heidelberg, 69120 Heidelberg, Germany

[3] Mechanobiology Institute, National University of Singapore, 117411 Singapore, Republic of Singapore

[4] Department of Biomedical Engineering, National University of Singapore, 117411 Singapore, Republic of Singapore

[5] School of Applied Chemistry, Reutlingen University, 72762 Reutlingen, Germany

[6] Internal Medicine I, University Clinic Ulm, 89081 Ulm, Germany

[7] General and Visceral Surgery, University Clinic Ulm, 89081 Ulm, Germany

[*]Corresponding author. Alteburgstrasse 150, 72762, Reutlingen, Germany. Tel: +49 7121 271-2070; E-mail address: ralf.kemkemer@reutlingen-university.de

Supplemental Video: Cell detachment during temperature sweep. HL-60 cells plated on glass exposed to a temperature sweep from 37-43 ºC before rapid return to 37 ºC. Images were obtained every 10 seconds for nearly 2.5 hours.

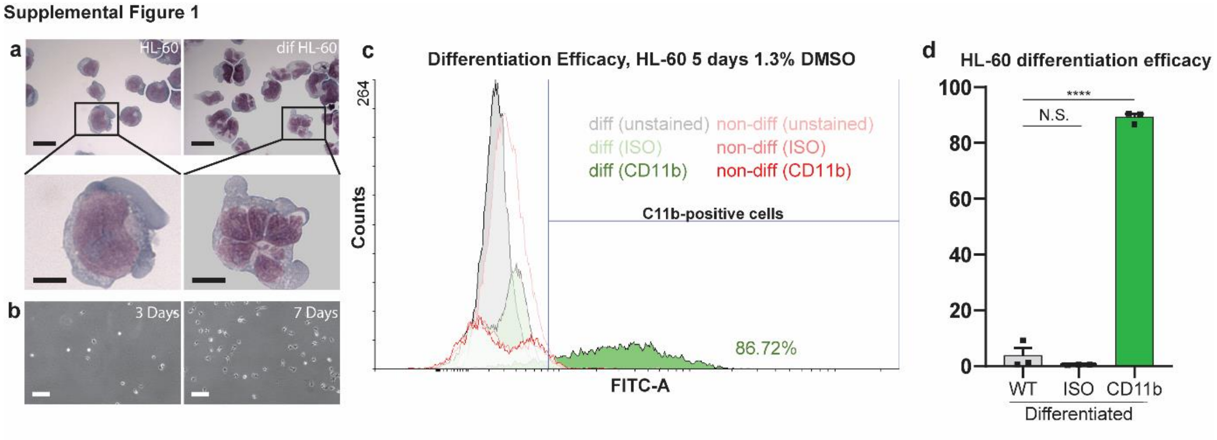

Supplemental Figure 1: Successful differentiation of HL-60 cells into neutrophil-like cells. **a.** Giemsa-Wright staining of HL-60 cells before (left) and 7 days after differentiation (right). Scale bars: 50 µm (top) and 20 µm (bottom). The majority of differentiated HL-60 cells exhibit lobed nuclei characteristic to blood PMN cells. **b.** 10x phase contrast images 3 days and 7 days after the start of differentiation. The amount of attached cells increases up to 10 fold. Scale bars: 50 µm. **c.** Flow cytometry of differentiated HL-60 cells after 5 days of 1.3% DMSO treatment. **d.** Differentiation efficiency of HL-60 cells as measured by CD11b expression.

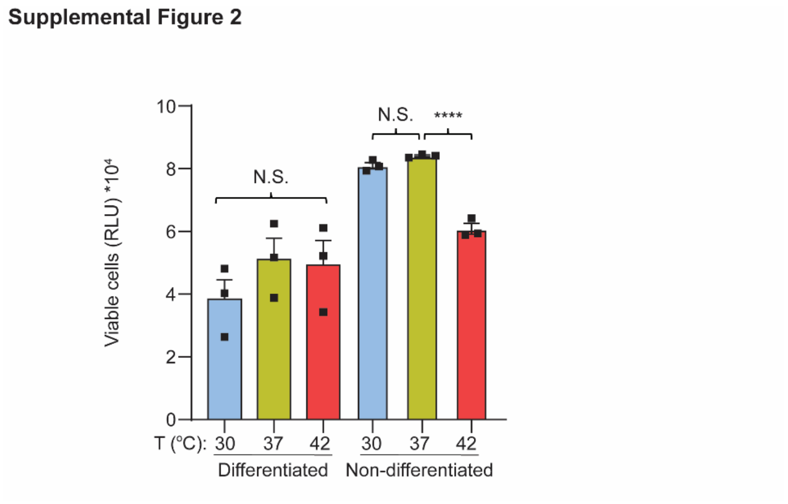

Supplemental Figure 2: HL-60 cell survival as a function of temperature. ATP-based cell viability determined with CellTiter-Glo assay to assess the relative level of viable cells at 30, 37, and 42 °C.

Supplemental Figure 3

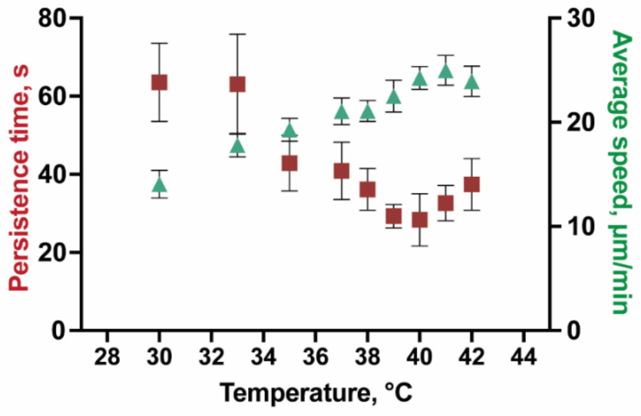

Supplemental Figure 3: Relationship between average cell speed and migration persistence time for differentiated HL-60 cells at control conditions. With increasing temperature cell average speed increases (right axis, green), but migration persistence time decreases (left axis, red).

Supplemental Figure 4

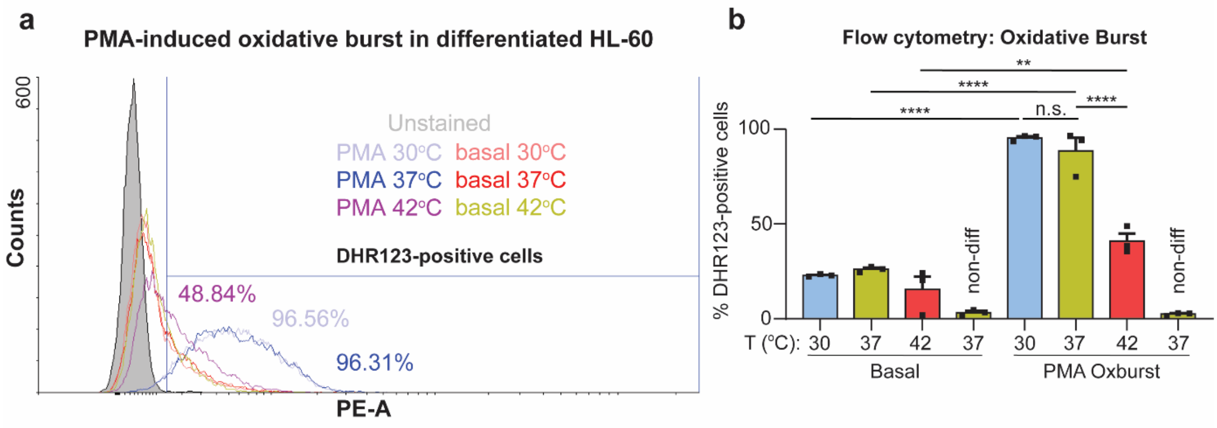

Supplemental Figure 4: Oxidative burst in differentiated HL-60 cells as a function of temperature.
a) Flow cytometry of PMA-induced oxidative burst in differentiated and non-differentiated HL-60 cells.
b) Quantitative analysis of the percentage of DHR123-positive cells with and without PMA induction.

Supplemental Figure 5

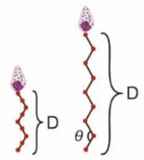

T	Low	High
$\bar{V}$	Low	High
P_L	=	=
P_t	Long	Short
D	Short	Long

Supplemental Figure 5: Higher speeds, lower persistence times, and equal persistence lengths allow cells to migrate more effectively at higher temperatures. An illustration of cell tracks at low and high temperatures. V is the average velocity, P_L is the persistence length, P_t is the persistence time and D is the length of the cell track. At low temperatures, cells have a long persistence time and short track lengths. At higher temperatures, cells have a short persistence time but long track lengths. As a result, persistence length stays the same at both low and high temperatures.
